# The Position of EGF Deprivation in the Management of Advanced Non-Small Cell Lung Cancer 

**DOI:** 10.3389/fonc.2021.639745

**Published:** 2021-06-15

**Authors:** Tania Crombet Ramos, Orestes Santos Morales, Grace K. Dy, Kalet León Monzón, Agustín Lage Dávila

**Affiliations:** ^1^ Research Direction, Center of Molecular Immunology, Havana, Cuba; ^2^ Department of Medicine, Roswell Park Comprehensive Cancer Center, Buffalo, NY, United States

**Keywords:** CIMAvax-EGF, NSCLC, EGFR, immune checkpoint inhibitors, tyrosine kinase inhibitors

## Abstract

Advanced non-small cell lung cancer (NSCLC) has faced a therapeutic revolution with the advent of tyrosine kinase inhibitors (TKIs) and immune checkpoints inhibitors (ICIs) approved for first and subsequent therapies. CIMAvax-EGF is a chemical conjugate between human-recombinant EGF and P64, a recombinant protein from *Neisseria meningitides*, which induces neutralizing antibodies against EGF. In the last 15 years, it has been extensively evaluated in advanced NSCLC patients. CIMAvax-EGF is safe, even after extended use, and able to keep EGF serum concentration below detectable levels. In a randomized phase III study, CIMAvax-EGF increased median overall survival of advanced NSCLC patients with at least stable disease after front-line chemotherapy. Patients bearing squamous-cell or adenocarcinomas and serum EGF concentration above 870 pg/ml had better survival compared to control patients treated with best supportive care as maintenance, confirming tumors’ sensitivity to the EGF depletion. This manuscript reviews the state-of-the-art NSCLC therapy and proposes the most promising scenarios for evaluating CIMAvax-EGF, particularly in combination with TKIs or ICIs. We hypothesize that the optimal combination of CIMAvax-EGF with established therapies can further contribute to transform advanced cancer into a manageable chronic disease, compatible with years of good quality of life.

## Introduction

Advanced non-small cell lung cancer (NSCLC) remains a major health problem. Despite all scientific advances of the last decades, a substantial fraction of lung cancer patients are diagnosed at advanced stages of disease, accounting for roughly 1.2 million deaths per year worldwide (1).

During the last decade, the high heterogeneity of advanced NSCLC has been confirmed (2, 3). Aside from histologic subtypes, molecular characterization of the disease is a critical step in the classification of the disease, making management of NSCLC much more complex (2–6). In this context, oncogene addiction to the epidermal growth factor receptor (EGFR) pathway is widely accepted (7, 8). Indeed, the dependence of some tumors on aberrant EGFR signaling supports the successful use of EGFR tyrosine kinase inhibitors (TKI) in tumors with known activating mutations (9, 10).

Epidermal growth factor (EGF) is one of the most important ligands of the EGFR, which is produced mainly in a paracrine fashion (11). EGF concentration in human serum is largely variable, both in healthy and lung cancer patients, but it tends to be higher in patients (12, 13). A high EGF amount plus EGFR overexpression in lung cancer cells (14), create conditions for the growth of some EGF-dependent tumors, even in the absence of specific EGFR driver mutations. Our group has shown that high EGF concentration in serum is a poor prognostic factor for advanced NSCLC individuals (15). CIMAvax-EGF is a growth factor-depleting immunotherapy intended to reduce EGF concentration, both in serum and in the tumor microenvironment (16–18).

Advanced NSCLC has recently faced a therapeutic revolution with the advent of new drugs registered for first and subsequent therapies (19, 20). The introduction of innovative treatments, particularly TKIs and immunotherapies, evolves so fast that the therapeutic landscape is constantly changing (21). In this setting, medical progress relies not only on the discovery of new drugs but also on the smart positioning of the novel medications among the other choices. In this complex scenario, we would like to discuss novel clinical trials with CIMavax-EGF, which could better potentiate other registered therapeutics.

## CIMAvax-EGF Immunotherapy

**Figure 1:**
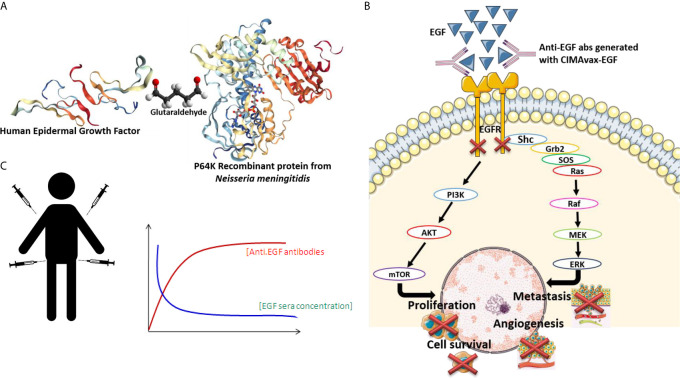
**(A)** Cimavax composition: CIMAvax-EGF is a chemical conjugate between human-recombinant EGF and P64, a recombinant protein from *Neisseria meningitides*. **(B)** CIMAvax-EGF induces neutralizing antibodies that trap EGF and reduces its concentration in serum, achieving an immunological “castration” of the growth factor. **(C)** Sera of patients immunized with CIMAvax-EGF block the binding between EGF and its receptor, inhibiting the EGFR phosphorylation and cell proliferation *in vitro*.

CIMAvax-EGF is a chemical conjugate between human-recombinant EGF and P64, a recombinant protein from *Neisseria meningitides* (16, 17) (**Figure 1A**). CIMAvax-EGF induces neutralizing antibodies that trap EGF and reduces its concentration in serum, achieving an immunological “castration” of the growth factor (**Figure 1B**). Indeed, sera of patients immunized with CIMAvax-EGF block the binding between EGF and its receptor, inhibiting the EGFR phosphorylation and cell proliferation *in vitro* (22) (**Figure 1C**)

In the last 15 years, CIMAvax-EGF has been extensively evaluated in advanced NSCLC patients. CIMAvax-EGF has proven to be safe, even after extended use, and capable to keep EGF serum concentration below detectable levels (23, 24). The anti-EGF antibody response did not cross-react with other EGFR ligands, such as TGF alpha and amphiregulin (22, 25). Notably, in some patients, TGF alpha augmented after 6 months of CIMAvax-EGF, while amphiregulin concentration did not change overtime (25). The increase of one or more ligands after effective EGFR blockade has been reported before (26, 27).

In a phase III randomized clinical trial conducted between 2008 and 2012 in 405 patients, CIMAvax-EGF increased the median overall survival (OS) of the advanced NSCLC patients that had at least stable disease after front-line chemotherapy (15). Median OS was 12.43 months in the vaccinated patients that completed induction *vs.* 9.43 months in the control arm (15). Survival advantage was larger in patients with high-serum EGF concentration. In patients with EGF levels >870 pg/ml, absolute survival gain was 5 months (15). Moreover, long-term survival rates were higher in vaccinated *vs.* control patients: 37% *vs* 20% (2-year survival rate) and 23% vs 0% (5-year survival rate) (15).

Other clinical and tumor characteristics have been associated with longer survival after CIMAvax-EGF (15). Notably, benefit was larger in patients with squamous cell carcinoma (HR 0.524) than in adenocarcinoma (HR 0.835), probably linked to the higher expression of wild type EGFR in the squamous histology (28). In addition, patients with a better immune status benefited more: OS was larger individuals with higher anti-EGF antibodies or lower markers of immune-senescence (29). The proportion of CD8^+^CD28^−^ T cells, CD4 T cells, and the CD4/CD8 ratio after chemotherapy correlated with the clinical benefit of CIMAvax-EGF. Vaccinated patients with CD4^+^ T cells counts greater than 40%, CD8^+^CD28^−^ T cells counts lower than 24% and a CD4/CD8 ratio > 2 after first-line platinum-based therapy, achieved a significantly larger survival, as compared to controls with the same phenotype (29). Other biomarkers associated with the inflammatory response (neutrophil to lymphocyte ratio, NLR) as well as the neutrophil and monocyte counts were useful to predict response to CIMAvax-EGF (30).

**Figure 2:**
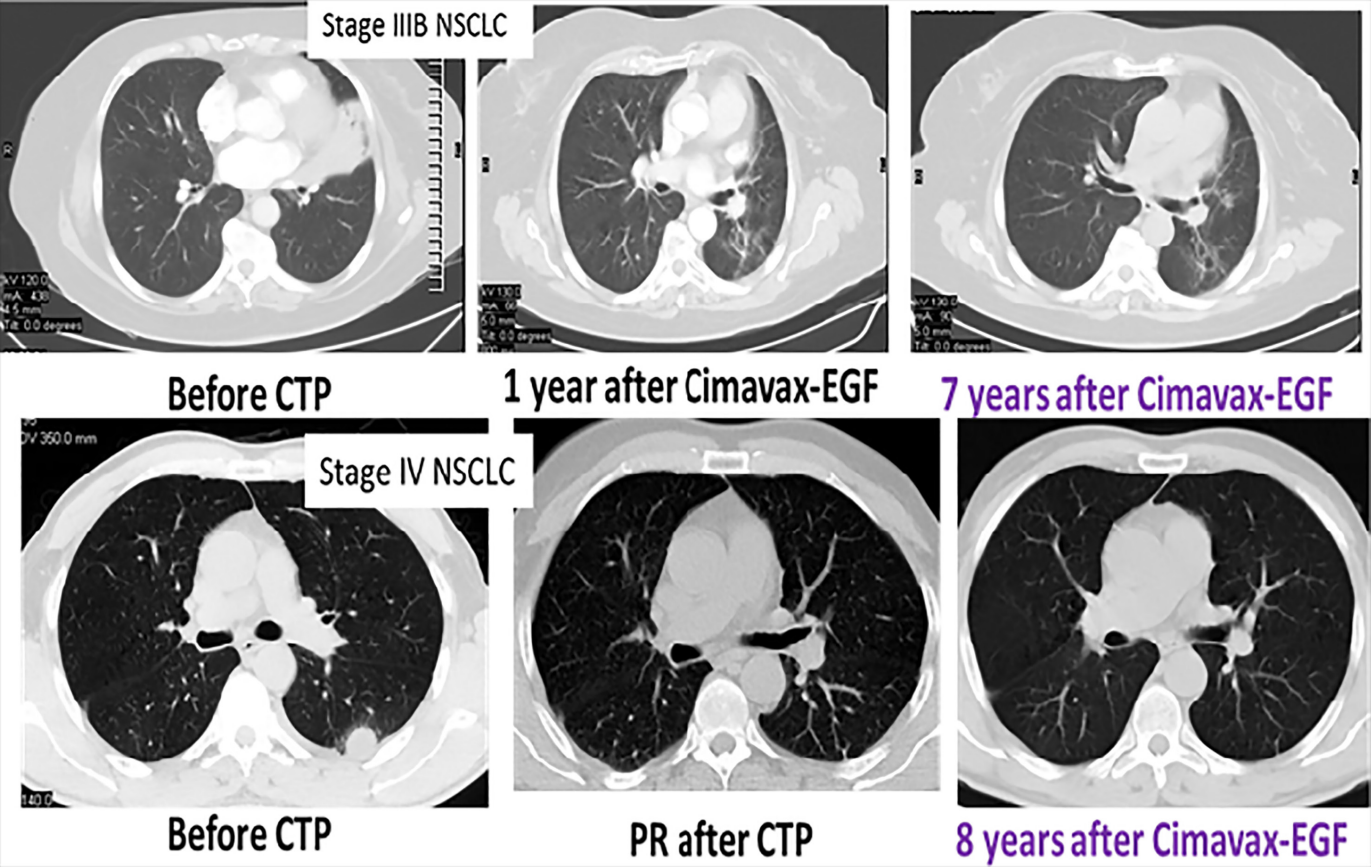
Long-lasting disease control after CIMAvax-EGF. CT scan series of two representative patients.

One of the key findings of the CIMAvax-EGF trials is the presence of a subgroup of patients with long-term survival, even in the absence of subsequent therapy (29, 31). These long-term survivors frequently exhibit a persistent but almost dormant or very slow-growth tumor (**Figure 2**), which resembles the behavior of prostate or breast tumors treated with hormone-depleting therapies (32, 33).

**Figure 3:**
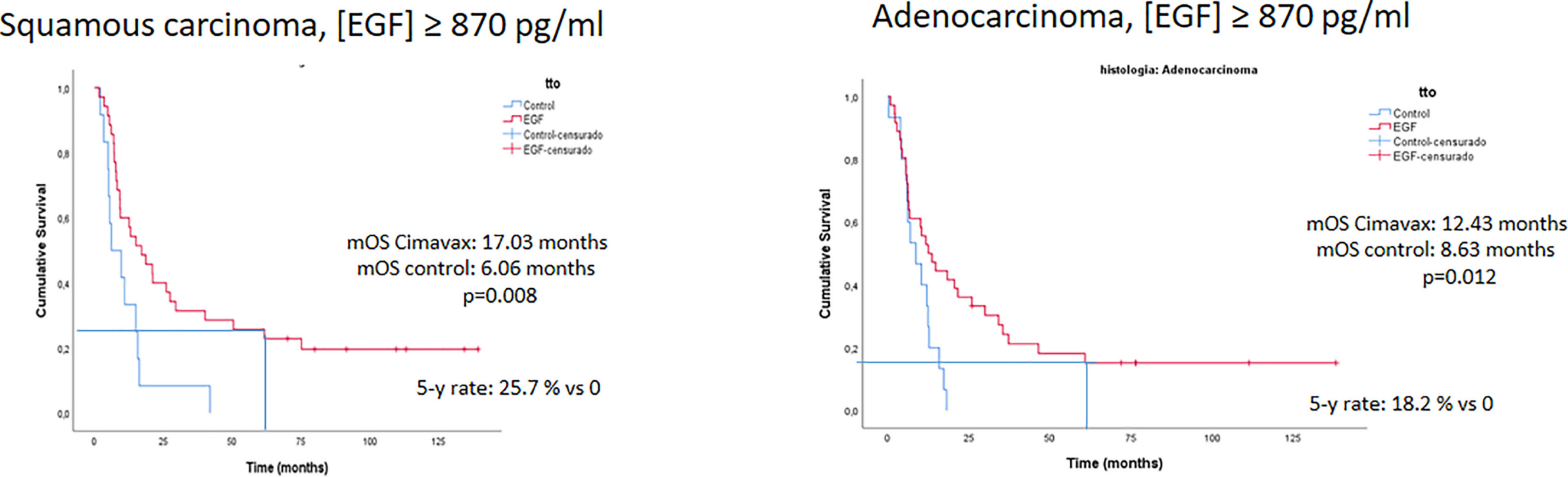
Survival advantage of EGF deprivation therapy over best supportive care according to serum EGF concentration and tumor histology: 5 years update of the phase III trial.

A recent update of the Cimavax-EGF Phase III clinical trial confirmed this previous finding. The 5-year survival rate was high in patients with adenocarcinoma or squamous cell carcinomas with serum EGF concentration above 870 pg/ml, confirming sensitivity of the tumors to the EGF depletion (**Figure 3**).

CIMAvax-EGF was initially approved as switch maintenance for all advanced NSCLC patients not progressing after first platinum-based chemotherapy. Later on, label was amended to include patient selection according the EGF concentration in serum.

### The Evolving Landscape of Advanced NSCLC Treatment

Over the past two decades, chemotherapy and, in particular, platinum-based combinations provided a modest survival advantage and symptom palliation for inoperable NSCLC patients (34). At the end of the 20th century, controlled clinical trials comparing doublet regimens (platinum plus taxanes, vinca alkaloids, or etoposide) found equal efficacy among treatment arms. Indeed, it seemed that a survival plateau (40% 1-year survival rate) was reached with traditional cytotoxic drug combinations (2).

In this context, efforts for obtaining an EGF-depleting therapy began. Epidermal growth factor was discovered in 1962, and the first clues on the role of EGF/EGFR in cancer cell biology appeared in the 1980s (35, 36). Several pieces of evidence showing the wide applicability of the cancer hormone-dependence concept to the emerging field of peptide growth factors came out from our team (37, 38).

CIMAvax-EGF treatment showed survival improvement as switch maintenance for NSCLC patients with disease control after platinum doublets. However, in parallel, two major scientific advances emerged, which led to radical changes in the standard treatment protocols for advanced NSCLC. These were the following:

1. The identification of genetic driver mutations (39) and the introduction of targeted therapies, such as tyrosine kinase inhibitors (TKI). TKIs are specific for mutated oncogenic proteins and have the advantage of being oral pills with reduced toxicity (40). Particularly, TKIs targeting specific EGFR and ALK/ROS1 mutations are widely used (41, 42).
2. The discovery of the immune “checkpoints,” which can be targeted with specific monoclonal antibodies (immune checkpoint inhibitors [ICI]). ICI lessens the control loops of the immune system, allowing the expansion of the antitumor immune response (43–45). The cytotoxic T lymphocyte–associated antigen 4 (CTLA-4) and programmed death 1 (PD-1) immune checkpoints are negative regulators of immune function. CTLA-4 regulates T-cell proliferation early in the immune response, mainly in lymph nodes, whereas PD-1 limits T cells later in an immune response, primarily in the peripheral tissues (46). Particularly, antibodies targeting programmed death molecule-1 (PD1) or their main ligands (PD-L1) are extensively used in the first-line setting (47).

**Figure 4:**
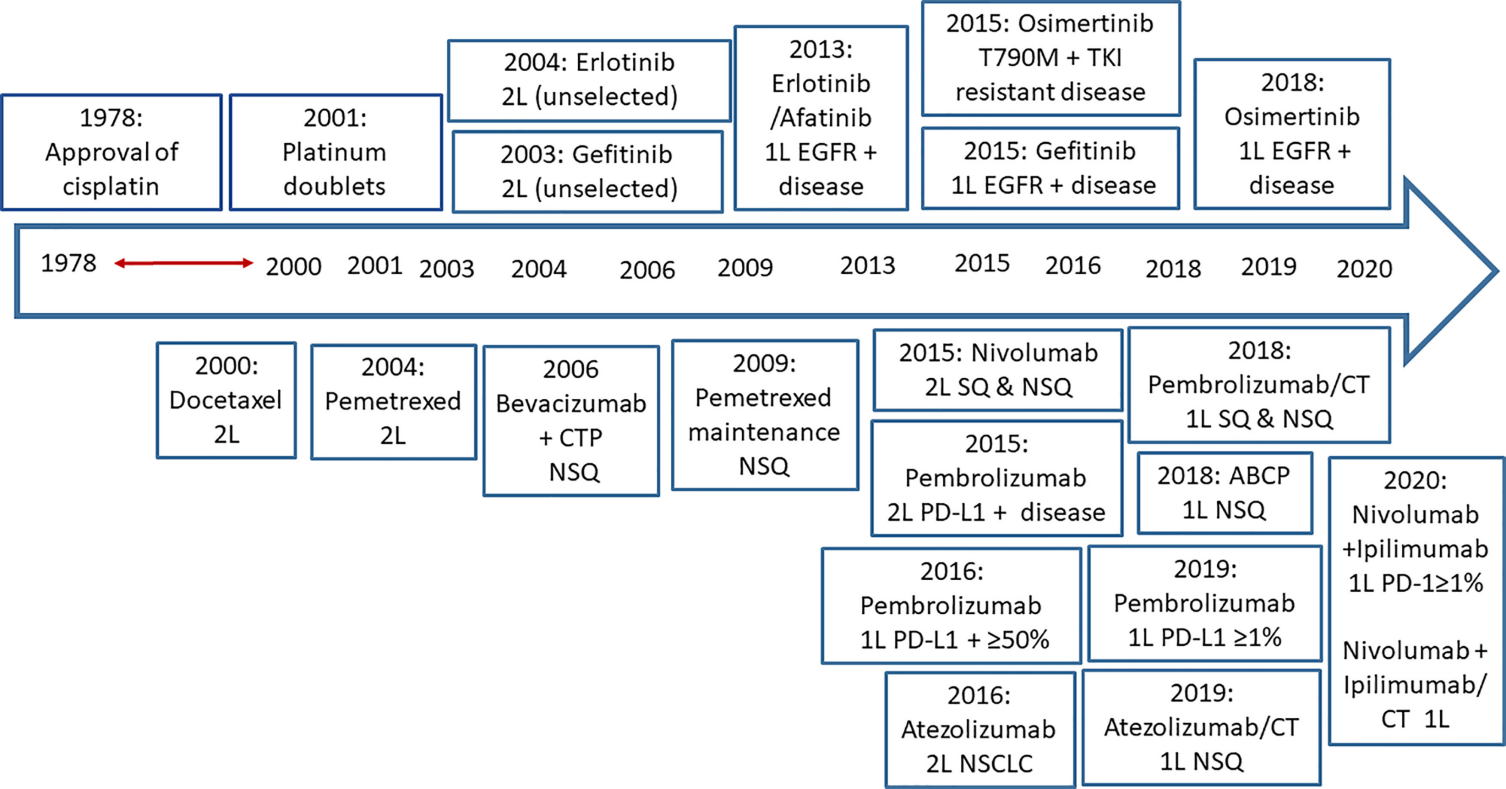
The evolving setting of advanced NSCLC therapy: From conventional chemotherapy to EGFR TKIs and checkpoint inhibitors. SOC, standard of care; 1L, first line; 2L, second line; SQ, squamous; NSQ, non-squamous; ABCP, atezolizumab-bevacizumab-carboplatin and paclitaxel.

The evolving landscape of advanced NSCLC therapy from conventional chemotherapy to EGFR TKIs and checkpoint inhibitors is depicted in **Figure 4**.

**Figure 5:**
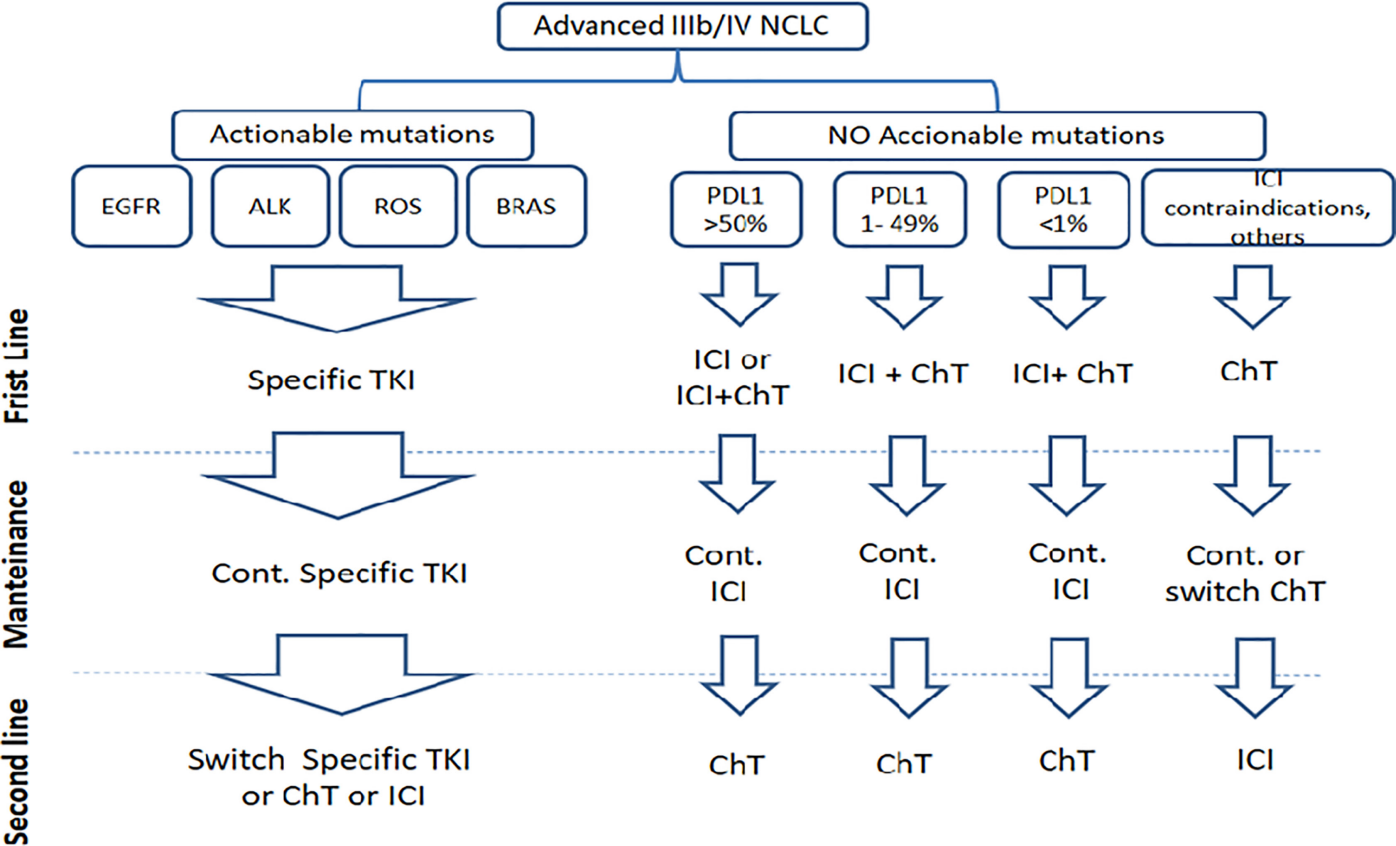
Current landscape of NSCLC according actionable mutations and PD-L1 expression.

Progressively, molecular stratification according to driver mutations (actionable mutations) and PD-L1 expression has gained preeminence, even over the classical histological classification. The identification of actionable mutations and the level of expression of PD-L1 have divided the current landscape of NSCLC into several therapeutic scenarios (**Figure 5**) apart from traditional chemotherapies. Moreover, the lower toxicities of the new drugs allow maintenance or even their use as consolidation therapies, such as after concurrent chemo-radiation in unresectable stage III NSCLC (48, 49). In the next sections, we briefly describe and comment the main therapeutic scenarios illustrated in **Figure 5**.

### Tumors With Actionable Mutations

Actionable mutations appear in roughly 40% to 50% of adenocarcinoma patients and are rare in squamous cell carcinomas (50). EGFR, ALK, ROS1, BRAF, MET, RET mutations are the most relevant for current lung cancer therapy, although many other mutations are still under active clinical investigation (51). The detection of such driver mutations determines the use of specific targeted therapy. In this manuscript, we focus on the current therapeutic alternatives for tumors bearing EGFR mutations (see **Table 1**).

**Table 1:** Therapeutics options for patients with actionable mutations of the EGFR, regardless histology.

Biomarker	Investigational drug	Response rate (months)	PFS months	OS months	1-Year survival rate	2-Year survival rate	Reference trial
First line therapy							
Mutated EGFR (exons 19 and 21)	Osimertinib	80%	18.9	38.6	89%	74%	FLAURA
	Gefitinib	71.2%	9.6	21.6	24.9 %	--	IPASS
	Erlotinib	58%	9.7	22.9	--	--	CALGB 30406
	Afatinib	56%	11.1	28.3	--	--	LUX-Lung 7
	Dacomitinib	75%	14.7	34.1	--	--	ARCHER 105
	Erlotinib+Ramucirumab	76%	19.4	--	93 %	83 %	RELAY
Second line therapy							
Mutated EGFR T790M	Osimertinib	71%	8.5	26.8	80%	55%	AURA-3

In patients with EGFR-mutated tumors, the use of specific TKI as first line has significantly increased the response rate and progression free survival (PFS) when compared with standard chemotherapy. First- and second-generation TKIs, such as gefitinib, erlotinib, afatinib, and dacomitinib, are used continuously until evidence of tumor resistance, which happen frequently by the selection of a secondary mutation in the kinase domain of the EGFR, particularly T790M (52). Osimertinib is a third-generation TKI, which is specific for EGFR mutations, including the T790M mutation. It has shown the best response rate and PFS observed so far in the first-line setting among patients with sensitizing EGFR mutations (53). For this patient population, subsequent therapy with cytotoxic drugs should be used only after exhausting all TKI possibilities. ICIs as initial or subsequent monotherapies are not recommended, since there is evidence of resistance of EGFR mutated tumors to the PD1/PD-L1 blockade, despite their frequently elevated PD-L1 expression (54, 55).

Combination of ramucirumab, an anti-VEGFR2 monoclonal antibody (MAb), with erlotinib has also shown significant PFS improvement as compared with single first- and second-generation TKI therapies in the first line (56). However, safety must be considered due to the higher frequency of grade 4 or serious adverse events (SAEs) observed with this combination (56). Recently, several preclinical and early clinical trials were performed to assess the combined effect of EGFR TKIs and PD1/PD-L1 targeting drugs. According Han and co-workers, treatment schedule would be crucial to increase the efficacy and safety of EGFR TKI and immunotherapy. ICIs can synergize better with hypo-fractionated TKI to achieve complete response of late-stage cancer. However, high-dose EGFR TKI can not only elicit a greater innate response but also increase the PD-L1 expression in tumors, contributing to resistance (57). According the initial trials, combination of TKIs and ICIs in patients with EGFR mutations could be toxic and had failed to show clinical benefit (58). Pembrolizumab plus erlotinib was feasible; however, pembrolizumab plus gefitinib provoked grade 3/4 liver toxicity in five of seven patients, leading to permanent treatment discontinuation in four individuals (58). In addition, PD-L1 blockade followed by osimertinib was associated with severe immune-related adverse events, particularly pneumonitis (59).

### Tumors With No Actionable Mutation

In tumors with unknown or no actionable mutations, ICIs blocking the interaction between PD-1 and PD-L1 ligand, ICI alone, or in combination with chemotherapy dominate the therapeutic landscape currently (**Figure 5**). Therapeutic interventions are largely guided by PD-L1 expression levels regardless of the classical NSCLC histological sub-classification. However, overall efficacy of the treatments and the specific choice of the chemotherapy regimen vary depending on histology.

### Tumors With PD-L1>50%

Tumors with high expression of PD-L1 represent 30% to 40% of all advanced NSCLC (60) and are highly sensitive to ICI provided there are no EGFR/ALK/ROS1 genetic alterations. Pembrolizumab, an anti-PD1 antibody, is the preferred first-line therapy for this type of tumors. This antibody can be used either alone or in combination with platinum-based chemotherapy based on the data from KEYNOTE 24, 42, 189, and 407 clinical trials (**Table 2**) (44, 60–63). However, an open debate remains in the literature regarding which patients should receive pembrolizumab alone *vs* its combination with chemotherapy (64, 65). Some authors suggest that clinical efficacy of pembrolizumab monotherapy is mainly demonstrated in patients with very high expression of PD-L1 (PDL-1>90%) (65). Atezolizumab has also shown benefits in comparison with chemotherapy as first-line monotherapy. The trial demonstrated a statistically significant improvement in OS for patients with high PD-L1 tumor expression (as defined by SP142 immunohistochemistry assay) receiving atezolizumab compared to those treated with platinum-based chemotherapy. Efficacy signals were very similar even with using DAKO 22C3 immunohistochemical stain to derive cutoff point of >/= 50% (used as companion diagnostic for pembrolizumab). Median OS was 20.2 months for patients in the atezolizumab arm compared with 13.1 months in the control arm (66).

**Table 2:** Most used therapeutic schemes in advanced NSCLC and no actionable mutations, according histology.

Biomarker	Investigational drug	Response rate	mPFS months	mOS months	1-Year survival rate	2-Year survival rate	Reference trial
**Frist-line therapy squamous cell carcinoma**							
PD-L1>50 %	Pembro+ChT	60.3%	8.0	NR	63.4%	–	KEYNOTE-407
1%<PDL-1<=49%	Pembro+ChT	49.5%	7.2	14.0	65.9%	–	KEYNOTE-407
PDL1<1%	Pembro+ChT	63.2%	6.3	15.9	64.2%	–	KEYNOTE-407
**Switch maintenance therapy squamous cell carcinoma**							
–	Docetaxel	11.7%	5.7	12.3	51.1%	–	Fidias, 2008.
[EGF]≥870 pg/ml	CIMAvax-EGF	–	–	17.03	60 %	40 %	
**Second-line therapy for squamous cell carcinoma**							
No previous ICI	Nivolumab	20%		9.2	23%	–	CHECKMATE-017
**First-line therapy non**–**squamous cell carcinoma**							
PD-L1>50 %	Pembro+ChT	62.1% (15.1)	11.1	NR >20.4	73.3%	51.9%	KEYNOTE-189
PD-L1>50 %	Pembro+ChT	62.1% (15.1)	11.1	NR >20.4	73.3%	51.9%	KEYNOTE-189
1%<PDL1<=49%	Pembro+ChT	49.2% (12.9)	9.2	21.8	71.7%	44.3%	KEYNOTE-189
PDL1<1%	Pembro+ChT	32.3% (10.8)	6.2	17.2	63.4%	38.5%	KEYNOTE-189
**Switch maintenance therapy non**–**squamous cell carcinoma**							
–	Pemetrexed	6.8%	4.5	15.5	--	--	Ciuleanu, 2009
[EGF]≥870 pg/ml	CIMAvax-EGF	–	–	12.43	52.8 %	36.1 %	
**Second-line therapy non**–**squamous cell carcinoma**							
No previous ICI	Nivolumab		2.3	12.2	50.5	29%	CHECKMATE-057

Other recommended combinations for non-squamous tumors consist of atezolizumab/carboplatin plus paclitaxel/bevacizumab or nab-paclitaxel and nivolumab/ipilimumab plus platinum/pemetrexed (64). The bevacizumab-containing combination was also the first and only regimen to date to demonstrate superior survival outcomes in patients with EGFR/ALK mutations who had disease progression on TKI prior to enrollment in the study. For squamous tumors, nivolumab/ipilimumab plus platinum/paclitaxel also seems to be efficacious (64). For both histologies, nivolumab + ipilimumab significantly prolonged progression-free survival *vs* chemotherapy in patients with high tumor mutational burden (67).

### Tumors With 1% < PD-L1 <= 49%

Tumors with intermediate expression of PD-L1 represent between 30% and 40% of all advanced NSCLC (63) and respond better to the combination of ICI and chemotherapy (64). For non-squamous, the best alternative is pembrolizumab + platinum/pemetrexed. Other accepted treatments are atezolizumab/carboplatin combined with paclitaxel/bevacizumab or nab-paclitaxel and nivolumab/ipilimumab plus platinum/pemetrexed. For squamous, the preferred choice is pembrolizumab/carboplatin plus paclitaxel or nab-paclitaxel. Nivolumab/ipilimumab plus carboplatin/paclitaxel can also be prescribed (64).

As in the previous subset, nivolumab and ipilimumab can be used in patients with high-mutation tumors.

### Tumors With PD-L1<=1%

Tumors with low or negative expression of PD-L1 represent roughly 30% of all advanced NSCLC (68), and they are not sensitive to ICIs alone. However, chemotherapy and ICI, as described in the previous subset, can be used for ECOG performance status 0-1 patients (64). The mechanistic explanation for this effect remains unclear, but it could reflect immune sensitization of the tumors by the applied chemotherapy.

### ICI Contraindicated, Patient Refusal, or Product Not Affordable

Despite PD-L1 expression level, several contraindications could prevent the use of ICI: active or documented history of autoimmune diseases, current use of immunosuppressive agents and ECOG > 2 (62, 63, 67, 69). Furthermore, there is a significant questioning in the literature regarding cost effectiveness of the combination of ICI + chemotherapy in PD-L1 low or negative tumors (70, 71). The high cost of ICIs in contrast to its clinical impact could lead to negative pharmacoeconomic evaluations, even for rich countries like US, Japan, or Europe, but definitely for many middle and low-income countries (72–75). Therefore, some national and/or private health insurances might choose not to cover ICI + chemotherapy in patients with PD-L1 < 1%, at least until a significant price reduction for ICIs takes place. Accordingly, a significant number of advanced NSCLC patients might not have access to ICIs in first-line or in even second-line setting. For this group of patients, traditional platinum-based chemotherapy (**Table 2**) remains the standard of care (76–78).

### Subsequent Therapy (Maintenance and Second Line)

In patients receiving ICI alone or in combination with chemotherapy in first-line, continuation maintenance with pembrolizumab alone or combined with pemetrexed (63, 68), atezolizumab alone, or combined with bevacizumab (69, 79) are the recommended schemes in the adenocarcinoma histology. For the squamous setting, pembrolizumab or atezolizumab monotherapies can be used as maintenance therapies (62, 66).

Prior to the advent of ICIs, switch or continuation maintenance chemotherapy is the standard of care (76–78). However, some concerns have been raised about the advantage of switch maintenance in advanced NSCLC due to the toxicity and modest survival benefit (76) (**Table 2**).

### CIMAvax-EGF Opportunities

There is an unquestionable clinical benefit of the newly adopted therapies and protocols for NSCLC (**Figure 5** and **Tables 1**, **2**). Indeed, beyond the statistical significance and differences in median survival, there was a modification of the shape of the survival curves. Cumulative survival curves of patients receiving ICIs did not show only better overall survival, but also a tail suggesting very prolonged survival and transition to chronicity of a subset of patients (63). Patients live longer with good quality of life (80). However, these undeniable improvements are still far from raising the bar of survival expectancies long enough in a larger fraction of patients to achieve substantial reduction in mortality rates, or major decreases in the mortality/incidence index, as already happened for hematologic malignancies, prostate and breast cancer (81, 82). The vast majority of lung cancer patients will eventually succumb from their disease (81). Therefore, the addition of CIMAvax-EGF and other new drugs that might further increase survival is warranted.

What could be the place of CIMAvax-EGF inside this new therapeutic landscape? Which strategies seem more promising for its rapid practical application and/or for designing new clinical trials?

The most straightforward positioning of CIMAvax-EGF is as switch maintenance in the subgroup of patients who are not candidates for ICI maintenance and have higher serum EGF levels. As explained, clinical trials with CIMAvax-EGF started prior to the adoption of mutation testing, targeted therapies, and ICI as standards. Under these circumstances, a clear survival advantage was demonstrated in advanced NSCLC patients treated with CIMAvax-EGF as switch-maintenance (**Figure 3** and **Table 2**). Particularly, a large benefit was seen in patients with high-serum EGF concentration, with a noteworthy 5-year survival rate of 18% and 26% for ADC and SCC histology, respectively (15) (**Figure 3**).

Given the low access to ICIs, the current standard for advanced NSCLC in Cuba for patients without actionable mutations and EGF concentration above 870 pg/ml consists of platinum doublets followed by switch maintenance with CIMavax-EGF. Moreover, given its low toxicity and the need of monthly re-immunizations, patients can receive booster vaccination at the primary care setting, under the supervision of trained family medicine physicians. However, this niche of patients will be contracted, both in Cuba and worldwide, as the access to ICIs increases.

Therefore, the larger opportunities of CIMAvax-EGF come from its potential use in combination with established therapies. In 2011, Hanahan and Weinberg proposed eight “hallmarks of cancer” (sustaining proliferative signaling, evading growth suppressors, resisting cell death, enabling replicative immortality, inducing angiogenesis, activating invasion and metastasis, reprogramming of cellular energy metabolism, and active evasion from attack by immune cells) in an attempt to explain the complexity of cancer biology (83). These hallmarks of cancer can become targets of therapeutic interventions and rational combinations.

EGF immune deprivation (with CIMAvax-EGF) targets the extracellular domain of EGFR, while TKIs recognize the intracellular cascade controlling cell proliferation and resistance to apoptosis. ICIs target the capacity of cancer cells to evade the immune system control. In the following sections, we debate potential combinations of CIMAvax-EGF with TKI or ICI in the treatment of advanced NSCLC.

### Combining CIMAvax-EGF and EGFR-TKI

**Figure 6:**
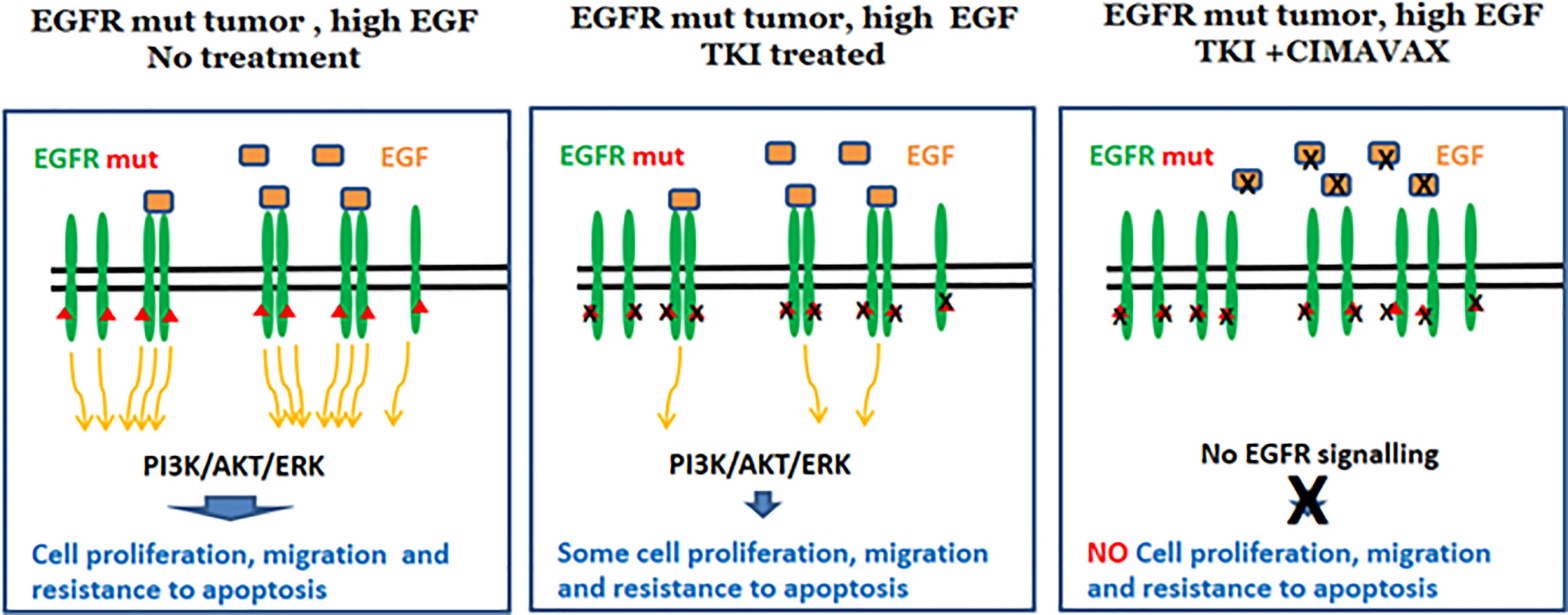
Rationale for CIMAvax-EGF combination with EGFR-TKI. Dual inhibition potentiates EGFR signaling inhibition.

For a long time, oncologists thought that tumors bearing EGFR-activating mutations in the intracellular domain were mostly independent from external ligand stimulation. However, recent *in vitro* studies showed that this might not be absolute. Codony-Servat et al. showed that EGF induces a basal signal on EGFR mutated tumors, even while treated with potent TKI, such as gefitinib, erlotinib, afatinib, and osimertinib (**Figure 6**) (84). Such basal EGFR signaling sustains slow tumor proliferation and survival, contributing to the escape of the cancer cells under TKI therapy. These *in vitro* observations explain previous findings of a worse response to TKI in patients with high serum levels of EGFR ligands, such as amphiregulin, TGFα, and EGF (85). Indeed, treatment of the EGFR mutant cell lines with anti-EGF polyclonal antibodies enhanced the antitumor activity of TKIs and delayed the appearance of resistant clones (84).

Overall, the latter findings provide a novel rationale for combining CIMAvax-EGF with EGFR TKI (**Figure 6**). Limited clinical data exist on the use of dual targeting of EGFR in patients with mutated NSCLC. Cetuximab, an anti-EGFR mAb, was combined with afatinib in a phase I study, in patients with acquired resistance to gefitinib/erlotinib. The combination of afatinib with cetuximab as second line of treatment demonstrated modest clinical activity with a median PFS of 4.7 months, a response rate of 29%, and 44% of grade 3 related adverse events (86). Combination treatment of afatinib and nimotuzumab demonstrated an acceptable safety profile and encouraging antitumor activity in advanced NSCLC patients with acquired resistance to gefitinib or erlotinib. The median PFS and overall survival were 4.0 and 11.7 months, respectively (87).

Potential caveats of this approach are safety concerns mainly associated with the use of high-affinity antibodies (cetuximab or panitumumab) and the lack of a strategy for selecting patients most likely to benefit. Therefore, the addition of CIMAvax-EGF to TKI is an appealing alternative, given CIMAvax-EGF excellent safety profile and the possibility of preselecting patients with high EGF concentration in serum (**Table 3**). Combination of CIMAvax-EGF in first line with TKI (EPICAL study) was launched in 2018. The trial will be completed in June 2021 (NCT03623750).

**Table 3:** Potential combination trials of CIMAvax-EGF plus EGFR TKIs or ICIs.

Treatment setting	Type pf patients	Drug combination	Main Endpoints
Stage III	Patients completing front-line	CIMAvax-EGF +	PFS
Maintenance	concurrent radio and chemotherapy	PD-L1 MAb	OS
Advanced or metastatic disease	Patients with EGFR-activating mutations	CIMAvax-EGF +	RR
First Line		EGFR TKI	PFS
			OS
Advanced or metastatic disease	Patients bearing tumors with	CIMAvax-EGF +	RR
First line	PD-L1 ≥ 50 %	anti-PD1/PD-L1 MAb	PFS
			OS
Advanced or metastatic disease	Squamous or Adenocarcinoma patients receiving	CIMAvax-EGF +	PFS
Maintenance	anti-PD1/PD-L1 monotherapy as maintenance	anti-PD1/PD-L1 MAb	OS
Advanced or metastatic disease	Patients not previously treated with anti-PD1/PD-L1 MAbs	CIMAvax-EGF+	OS
Second line		anti-PD-1/PD-L1MAbs	PFS
			DCR

### Combining CIMAvax-EGF and ICI

**Figure 7:**
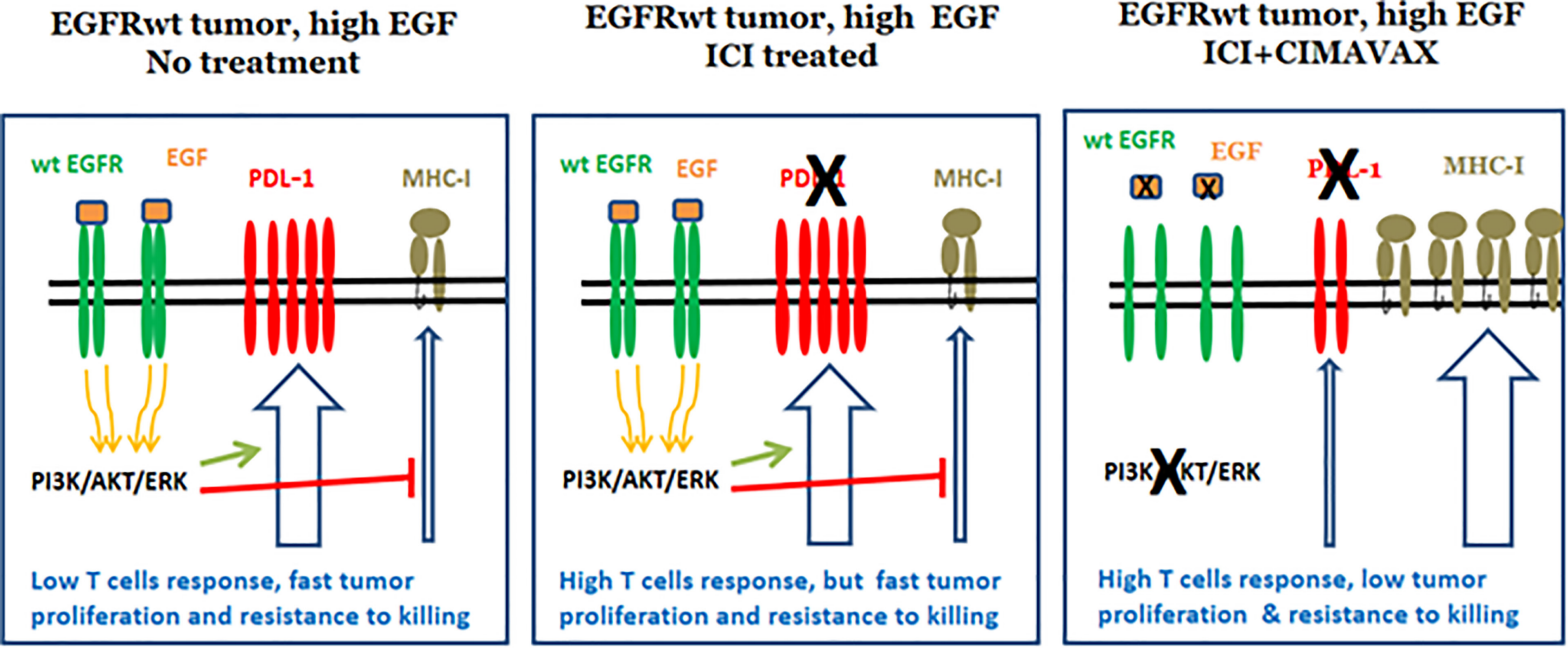
Reduction of PD-L1 expression after EGF depletion.

Immune evasion and growth factor dependence are two hallmarks of cancer, which can be targeted by ICI and CIMAavax-EGF or anti-EGFR MAbs. However, these two properties are clearly connected at the molecular level. EGFR activation has been associated with a down regulation of MHC-I and the antigen processing machinery (**Figure 7**), making tumor cells less sensitive to the immune system attack (88). For instance, treatment of cancer cells lines with the anti-EGFR MAb nimotuzumab *in vitro* induced the upregulation MHC class I expression (89). EGFR activation has also been associated with the induction of an immunosuppressive environment by the tumor cells, *via* up-regulation of pro-inflammatory cytokine secretion (90, 91). Transcription factors, like Stat3, NF-Кβ, and HIF-1α, link oncogene activation signaling pathways with these molecular mechanisms (92). Therefore, the use of ICI on tumors with a strong active signaling *via* EGFR might be less effective, given that the cancer cells impair, at least partially, the therapeutic effect of the activated CD8 T cells (**Figure 7**).

Furthermore, compelling evidences on the relationship between EGFR and PD-L1 expression in tumors are progressively accumulating (93–95). EGFR activation, through the interaction with natural ligands or by the existence of activating mutations, upregulates the expression of PD-L1 in cancer cells *in vitro* (96). Moreover, in lung cancer patients, there is a positive correlation between EGFR and PD-L1 expression (97). Therefore, the inhibition of EGFR signaling might reduce PD-L1 expression in the tumor cells, further reducing PD-L1–mediated tumor evasion. This effect enhances the effectiveness of the CD8 T cell response potentiated by ICI treatment (**Figure 7**). ICI treatments would inhibit PD1-PD-L1 interaction in the tumor cells, but also in the antigen presenting cells inside the tumor and lymph nodes, further promoting CD8 T cells activation.

Overall, the latter findings provide a novel rationale for the combination of CIMAvax-EGF with ICI (**Figure 7**). Combining checkpoint inhibitors and EGF deprivation would restore the immune system activation, presumably with higher sensitivity as a result of the EGFR signaling inhibition. Moreover, EGFR inhibition shall have a direct antitumor effect mediated by the blockade of the tumor proliferation and apoptosis induction.

Recently, the first results of combining an anti-EGFR antibody with an ICI in advanced NSCLC were reported. Necitumumab, an anti-EGFR mAb, was combined with pembrolizumab in 64 stage IV NSCLC patients who had progressed after platinum-based doublet, irrespective of PD-L1 expression and histology. The authors argue a manageable safety with SAEs in 42% of the patients. Median PFS was 4.1 months, and OS at 6 months was 74.7% (98) (**Table 2**).

CIMAvax-EGF is able to inhibit EGFR-mediated signaling without the well-recognized toxicities associated with the use of high affinities anti-EGFR mAb (15, 31, 99). Thus, it offers an attractive alternative to produce a synergistic effect between EGFR signaling inhibition and PD1 blockade without major toxicity concerns (**Table 3**). Below we discuss three different scenarios to do so:

### Combination of CIMAvax-EGF and Nivolumab in Second Line

A phase I/II clinical trial combining CIMAvax-EGF with nivolumab, as second line in NSCLC is open and recruiting patients at the Roswell Park Comprehensive Cancer Center (NCT02955290). Safety profile was good with no significant toxicities added by CIMAvax-EGF to the anti-PD1. A fast induction of anti-EGF antibodies and a reduction of EGF concentration in patient’s serum were also observed (100). Remarkably, overall response rate was 30% among PD-L1–negative patients and a median OS of 22.4 months was reported for patients with wild type KRAS (101). These figures compare well with those reported for nivolumab alone as second-line treatment (102).

However, this specific niche of patients (ICI second line, **Figure 5**) will be progressively reduced as the use of ICIs in first line becomes widely implemented. Therefore, there is a need to relocate the combination of CIMAvax-EGF with ICI in the context of first-line or maintenance scenarios.

### Combination of CIMAvax-EGF and ICI in Maintenance

CIMAvax-EGF can be used for prolonged periods as maintenance treatment without cumulative toxicity (31). This finding could support the use of CIMAvax-EGF in the maintenance setting, with pembrolizumab or atezolizumab in the squamous scenario (**Table 3**). This strategy can also apply to adenocarcinoma patients that would receive maintenance therapy with checkpoint inhibitors alone. This could be the case of patients with high PD-L1 expression, receiving chemo-immunotherapy in the front line, but maintenance therapy with ICI monotherapy. Preselection of patients with high EGF concentration and KRAS-wild type would be highly recommended. This combination shall increase response duration and PFS, increasing the proportion of long-term survivors.

### Combination of CIMAvax-EGF and ICI in First Line

Other alternative is to position CIMAvax-EGF in combination with pembrolizumab or atezolizumab, as starting therapy in stage IV NSCLC with 50% overexpression of PD-L1, irrespective of histology (**Table 3**). Preselecting patients with high EGF serum concentration and KRAS wild type will be crucial to potentiate combination. The benefit of using the combination as first line shall increase objective response as well as response duration and PFS.

### Combination of CIMAvax-EGF and Durvalumab in Stage III Tumors

The last alternative would be to combine CIMAvax-EGF with durvalumab in stage III NSCLC after completing front-line concurrent radio and chemotherapy (**Table 3**). In this scenario, median progression-free survival from randomization was 17.2 months with durvalumab versus 5.6 months with placebo (48). We hypothesize that combining CIMAvax-EGF with the anti-PDL1 can further increase PFS and overall survival in this patient subset.

## Discussion

CIMAvax-EGF is a new class of therapy aiming to induce antibodies against self-growth factors. It targets the EGF-EGFR pathway, a validated target on tumorigenesis. However, its larger impact will depend on the smart insertion of immune EGF deprivation into the complex algorithm of lung cancer management. Exploring the overlapping zone between interventions in the control of cell proliferation and immune evasion should be a priority of the current therapeutic algorithms.

Understanding the therapeutic potential of CIMAvax-EGF will demand a series of clinical trials devoted to identify the highest impact niches, potentiation with other treatment, optimal schedules, and new biological markers. The potential role of CIMAvax-EGF in increasing the clinical response achieved with other treatments like TKI or ICI is particularly attractive. Alternatively, CIMAvax-EGF can also help subgroups of patients who are not benefiting from the current therapies, like PD-L1 negative or EGFR wild type individuals.

In summary, CIMAvax-EGF is a therapeutic approach that has already been proven to be safe and efficacious, mainly in patients with high EGF levels and wild-type KRAS. We hypothesize that the smart combination of CIMAVax-EGF with the established EGFR TKI and ICIs can further contribute to the transition of advanced cancer to a chronic disease, compatible with years of quality life.

## Author Contributions

All authors contributed to the article and approved the submitted version.

## Funding

This research was funded by the Center of Molecular Immunology and the Cuban Ministry of Health.

## Conflict of Interest

Authors TC, OS, KL, AL currently work for the Center of Molecular Immunology, the institution that generated and originally patented CIMAvax-EGF.

The remaining author declares that the research was conducted in the absence of any commercial or financial relationships that could be construed as a potential conflict of interest.
